# Quantifying the heterogeneous impact of lockdown policies on different socioeconomic classes during the first COVID-19 wave in Colombia

**DOI:** 10.1038/s41598-023-43685-8

**Published:** 2023-09-30

**Authors:** Pablo Valgañón, Andrés F. Useche, David Soriano-Paños, Gourab Ghoshal, Jesús Gómez-Gardeñes

**Affiliations:** 1 Departament of Condensed Matter Physics, University of Zaragoza, 50009 Zaragoza, Spain; 2 GOTHAM lab, Institute for Biocomputation and Physics of Complex Systems, University of Zaragoza, 50018 Zaragoza, Spain; 3 Department of Industrial Engineering, School of Engineering, Universidad de Los Andes, 111711 Bogotá, Colombia; 4 Instituto Gulbenkian de Ciência, 2780-156 Oeiras, Portugal; 5 Department of Physics and Astronomy, University of Rochester, Rochester, NY 14627 USA

**Keywords:** Complex networks, Computer science

## Abstract

In the absence of vaccines, the most widespread reaction to curb the COVID-19 pandemic worldwide was the implementation of lockdowns or stay-at-home policies. Despite the reported usefulness of such policies, their efficiency was highly constrained by socioeconomic factors determining their feasibility and their associated outcome in terms of mobility reduction and the subsequent limitation of social activity. Here we investigate the impact of lockdown policies on the mobility patterns of different socioeconomic classes in the three major cities of Colombia during the first wave of the COVID-19 pandemic. In global terms, we find a consistent positive correlation between the reduction in mobility levels and the socioeconomic stratum of the population in the three cities, implying that those with lower incomes were less capable of adopting the aforementioned policies. Our analysis also suggests a strong restructuring of the mobility network of lowest socioeconomic strata during COVID-19 lockdown, increasing their endogenous mixing while hampering their connections with wealthiest areas due to a sharp reduction in long-distance trips.

## Introduction

During the first half of 2020, the lack of approved vaccines turned non-pharmaceutical interventions^1–4^ (NPIs) into our best ally to fight the first wave of the COVID-19 pandemic worldwide. NPIs encompass a wide variety of policies implemented to reshape the interaction patterns of the population to hamper the potential contagion pathways of infectious individuals. This goal can be accomplished by targeting different factors involved in virus transmission. At the individual level, the transmissibility of a virus in contacts between the infected and susceptible population is reduced by promoting prophylactic measures such as the use of masks^5,6^. At the community level, the exposure of susceptible individuals to a virus can be reduced by testing-trace-isolate-quarantine (TTIQ) policies^7–9^, shortening the effective infectious window of infectious individuals and isolating their contacts, or by population wide lockdowns and stay-at-home policies, decreasing the number of acquaintances of the entire population by controlling their mobility^10–12^.

The success of community NPIs, such as lockdown or stay-at-home policies, in reducing social contacts is not universal but strongly depends on the complex relationship existing between the socioeconomic characteristics of the population and their mobility^13^. In this sense, several studies have found remarkably different mobility patterns across socioeconomic classes classified according to several criteria such as race, ethnicity or income level^14–19^. In the context of epidemics, it is worth remarking that, even in uncontrolled scenarios, the coexistence of multiple mobility networks might be highly relevant for the evolution of epidemic outbreaks, leading to heterogeneous epidemic trajectories across the different socioeconomic classes^20,21^. In addition to the socioeconomic flavor of daily recurrent mobility patterns, the feasibility of the aforementioned policies also varies across different socioeconomic groups. For example, the adoption of stay-at-home policies is clearly related to the possibility of working at home, an option that is much more accessible to those individuals with higher income levels^22–24^. Consequently, a positive correlation between the income level of the population and the levels of mobility reduction achieved through lockdown interventions has been measured in different cities^25,26^.

Here we aim at further investigating the interplay between socioeconomic features and the mobility reduction observed as a consequence of lockdown policies in the three major cities of Colombia: Bogotá, Medellín and Santiago de Cali. As usual in Colombian cities, the population is divided into six different socioeconomic strata according to the quality of their households and neighborhoods. This classification is used to determine the cost of public services and access to government aid. Although there is not a one-to-one correspondence between strata and socioeconomic classes, stratum 1 typically gathers those individuals with less economic resources whereas stratum 6 usually correspond to the wealthiest population. The distribution of the population across strata is not homogeneous. For instance, local surveys carried out in Bogotá in 2017^27^ revealed that $86\%$ of the population belongs to the strata 1–3 whereas the highest strata, i.e., strata 5–6 only gather $4.5\%$ of its population. Similarly, the spatial distribution of these strata is not homogeneous either. For instance, in Bogotá, socioeconomic strata are highly spatially segregated, being the lowest one located to the south of the city^28,29^, a poorly connected area with an inadequate service infrastructure and deficiencies in access to health systems. This pattern is consistent across the other cities here studied, where lower socioeconomic strata are located far from the city centers, with conditions similar to those described in the case of Bogotá^29–31^.

Previous studies have already studied the heterogenous impact of lockdown policies on the mobility of different socioeconomic strata in Bogotá. In particular, Dueñas et al.^32^ analyze time variations in the use of public transport, finding higher mobility reductions for the wealthiest socioeconomic strata and a generalized shrinkage of trips distance. Here we use another source for mobility data, which are estimated from the travel patterns of mobile phone users, and extend the analysis to more Colombian cities. The spatio-temporal resolution of this dataset allows us to construct a time-varying multiplex network containing weekly mobility patterns for each stratum. We analyze the properties of such network, providing further evidence of how socioeconomic constraints shaped the outcome of lockdown policies in Colombia. Finally, we go one step further and analyze how lockdown policies altered the structure of the mobility patterns of different strata and their social mixing. By integrating both socioeconomic and mobility data, we reveal that lockdown policies also shaped the social structure of cities, exacerbating the segregation of lowest socioeconomic strata while barely affecting the mobility patterns of the highest ones.

## Methods

### Mobility dataset

The mobility patterns of the population are sourced from The Google COVID-19 Aggregated Mobility Research Dataset. The dataset contains anonymized mobility flows aggregated over mobile phone users who have turned on the Location History setting, which is off by default. This is similar to the data used to show how busy certain types of places are in Google Maps—helping identify when a local business tends to be the most crowded. The data set aggregates weekly flows of people from region to region, with a spatial resolution of level 12 s2cells areas. These correspond to an area between 3.04 and 6.38 squared kilometres. By default, all metrics defined in this section correspond to weekly values, but can be generalized to quantify averages over any arbitrary number of weeks.

To produce this data set, machine learning is applied to logs data to automatically segment it into semantic trips^33^. To provide strong privacy guarantees, all trips were anonymized and aggregated using a differentially private mechanism^34^ to aggregate flows over time (see https://policies.google.com/technologies/anonymization). This research is done on the resulting heavily aggregated and differentially private data. No individual user data was ever manually inspected, only heavily aggregated flows of large populations were handled.

All anonymized trips are processed in aggregate to extract their origin and destination location and time. For example, if users traveled from location a to location b within time interval t, the corresponding cell (a,b,t) in the tensor would be $n\pm err$, where *err* is Laplacian noise. The automated Laplace mechanism adds random noise drawn from a zero mean Laplace distribution and yields ($\varepsilon , \delta$)-differential privacy guarantee of $\varepsilon =0.66$ and $\delta = 2.1 \times 10-29$ per metric. Specifically, for each week W and each location pair (A,B), the number of unique users who took a trip from location A to location B during week W is computed. To each of these metrics, Laplace noise is added from a zero-mean distribution of scale 1/0.66. All metrics for which the noisy number of users is lower than 100 are then removed, following the process described in^34^, and the rest published. This yields that each published metric satisfies ($\varepsilon$,$\delta$)-differential privacy with values defined above. The parameter $\varepsilon$ controls the noise intensity in terms of its variance, while $\delta$ represents the deviation from pure $\varepsilon$-privacy. The closer they are to zero, the stronger the privacy guarantees.

### Demographic and socioeconomic data

We extract demographic and socioeconomic data from the 2018 Colombian census. Specifically, we use the spatial distribution of the population and the economic stratum of households aggregated to the level of census block. This way, for each census block *k*, we obtain the number of residents $\tilde{n}_k$ and the fraction of households belonging to each stratum *s*, $E^s_k$.

### Construction of the mobility multiplex network

To construct the mobility networks associated to each stratum, we should aggregate both socioeconomic and demographic information to match the spatial resolution of mobility data, corresponding to level 12 s2cells areas as discussed above. To do this, we associate each census sector with the cell containing its centroid. Let us here remark that Colombian cities typically display high levels of segregation among socioeconomic classes^15^, which allows us not to lose detail or aggregate disparate population when merging mobility and socioeconomic data. After this process, the number of residents inside cell *i* from stratum *s* is computed as:

1 $$\begin{aligned} n_i^s = \sum \limits _{k\in i} E_k^s \tilde{n}_k \;. \end{aligned}$$

From these quantities, we can straigthforwardly obtain the proportion of individuals belonging to a given stratum *s* in a cell *i*, $R_i^s$,which reads:

2 $$\begin{aligned} R_i^s = \dfrac{n_i^s}{\sum \limits _{s=1}^6{n_i^s}}\;. \end{aligned}$$

Finally, we assume that there are no significant differences in the representativeness of each stratum in the mobility data set. The latter assumption allows us to express the flow of residents from stratum *s* moving from cell *i* to cell *j* at a given week *t*, $f^s_{ij}(t)$, as

3 $$\begin{aligned} f_{ij}^s(t)= F_{ij}(t) R_i^s\ , \end{aligned}$$

where $F_{ij}(t)$ represents the origin-destination fluxes estimated from mobility data during week *t*. In what follows, we omit the time index in the explanation to ease notation. The sum of these trips across origins and destinations gives us the overall mobility of the population of stratum *s* during a week, which reads as follows:

4 $$\begin{aligned} f^s = \sum _{i,j} f_{ij}^s\;. \end{aligned}$$

Likewise, for the analysis of the outward mobility at each patch, it is only necessary to sum over the destinations as

5 $$\begin{aligned} f_i^s = \sum _{j} f_{ij}^s\;. \end{aligned}$$

Conceptually, for each week analyzed here, each city is thus represented by a multiplex mobility network **f** with $L=6$ layers, each one associated with a different socioeconomic stratum, *N* patches, corresponding to the different s2cells in which each city is partitioned in the mobility data set. For the baseline scenario used to compute the effects of lockdown policies, we consider the mobility patterns recorded for the week starting on 02-02-2020.

### Estimating mixing patterns among strata

Once we have embedded the mobility patterns of the different strata in a multiplex network, we can estimate how mixing between different strata changed over time as a consequence of the implemented lockdown policies. To this aim, we define the entries of the ($6\times 6$) mixing matrix $\textbf{M}$, for which each entry $M_{s_1 s_2}$ quantifies the average proportion of individuals belonging to stratum $s_2$ in all destinations visited by individuals from stratum $s_1$. Mathematically we compute these entries as:

6 $$\begin{aligned} M_{s_1 s_2} = \dfrac{\sum \limits _{i,j} f_{ij}^{s_1} R_j^{s_2}}{\sum \limits _{ij} f_{ij}^{s_1}}\;. \end{aligned}$$

To compare this metric with the mean-field baseline scenario, in which the population would be well-mixed, we divide these values by the fraction of the population belonging to each stratum. This is a more representative way of analyzing the segregation of the different strata without the bias of the underlying heterogeneous distribution of the population across strata. The entries of the re-normalized mixing matrix, $\mathbf{M_N}$, are thus defined as:

7 $$\begin{aligned} {M_N}_{s_1 s_2} = \dfrac{M_{s_1 s_2}}{n^{s_2}} \end{aligned}$$

where $n^{s_2}$ is the total population that belongs to stratum $s_2$ such that

8 $$\begin{aligned} n^s = \frac{\sum \limits _{i}n_i^{s}}{\sum \limits _{i,s'} n_i^{s'}}\;. \end{aligned}$$

Each row of the mixing matrix provides information about the segregation of the stratum $s_1$, which we can quantify in a single metric using Shannon entropy formula. This value, denoted by $S(s_1)$ is larger the less segregated the stratum is, as the components of the row become more homogeneous. We compute it as:

9 $$\begin{aligned} S(s_1) = - \sum _{s_2} M_{s_1 s_2} \ln {(M_{s_1 s_2})}\;. \end{aligned}$$

Another metric providing useful information about how people from different strata mix is the average distance that any person needs to travel to reach her destination. This distance can change over time and depends on the stratum of the individual. In particular, the average distance an individual from stratum *s* travels, denoted in what follows by $\langle {d^s}\rangle$ is calculated as:

10 $$\begin{aligned} \langle {d^s}\rangle = \dfrac{\sum _{ij} d_{ij} f_{ij}^s}{\sum _{ij}f_{ij}^s}\,, \end{aligned}$$

where $d_{ij}$ is the geometric distance between the centroids of geographical areas *i* and *j*. Analogously, the distance a person from stratum $s_1$ travels on average to arrive at a stratum $s_2$ destination, hereinafter denoted by $D^{s_1s_2}$, is computed as

11 $$\begin{aligned} D^{s_1 s_2} = \dfrac{\sum \limits _{i,j} d_{ij}f_{ij}^{s_1}R_j^{s_2}}{\sum \limits _{i,j} f_{ij}^{s_1} R_j^{s_2} }\;. \end{aligned}$$

## Results

**Figure 1:**
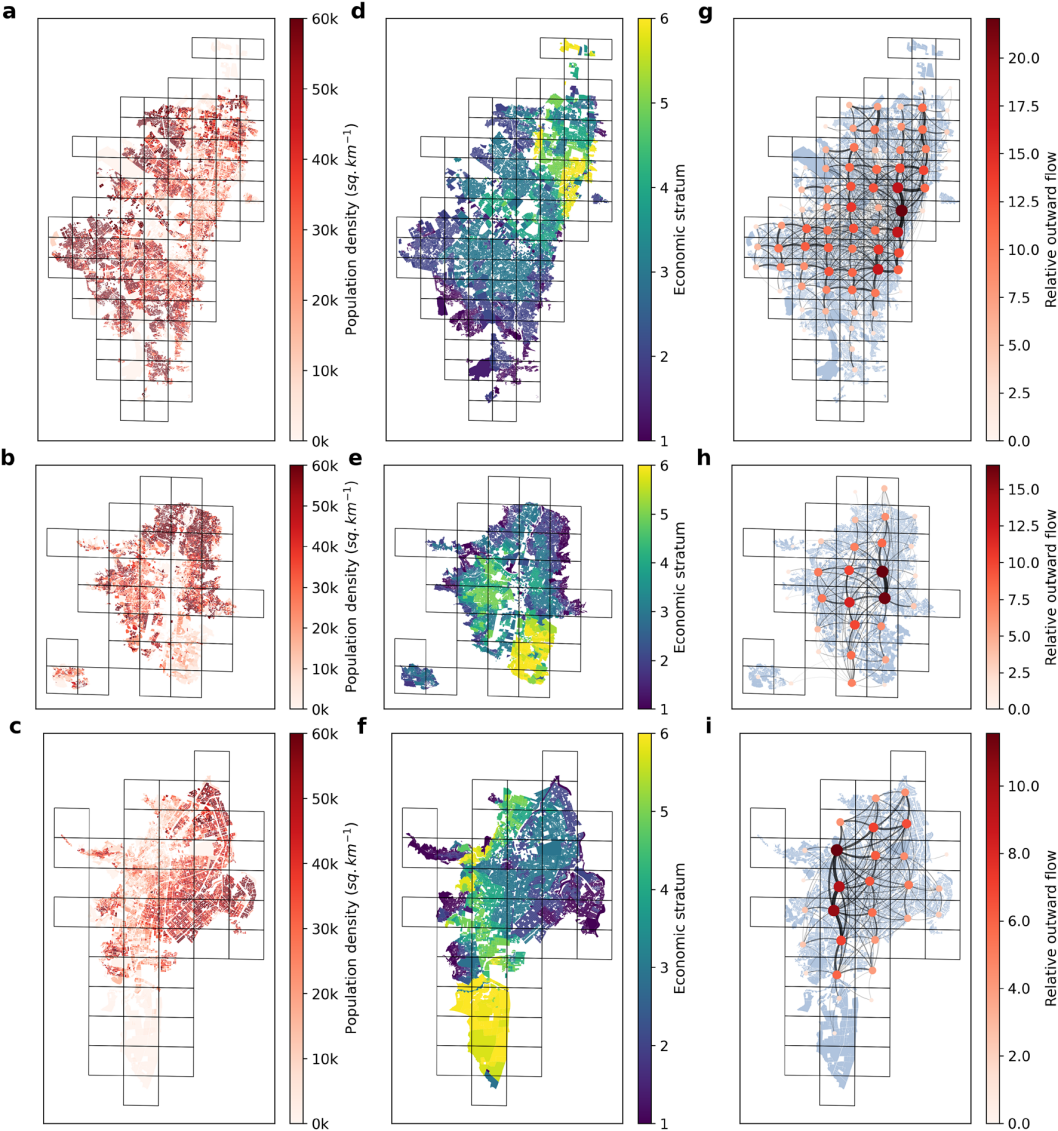
(**a**–**c**) Population density maps at the level of census block, corresponding to the 2018 census. (**d**–**f**) Average economic strata of the different households at the level of census block, ranging from stratum 1 typically gathering those individuals with lowest economic income to stratum 6 associated with wealthiest population. (**g**–**i**) Schematic representation of the mobility network of each city with a spatial resolution of level 12 s2cells (see “Methods” for further explanations). Both color and size of nodes are proportional to the total number of trips departing from a given area whereas the edge thickness reflects the the number of trips recorded between two locations. From top to bottom, the information shown corresponds to Bogotá, Medellín and Santiago de Cali respectively.

**Figure 2:**
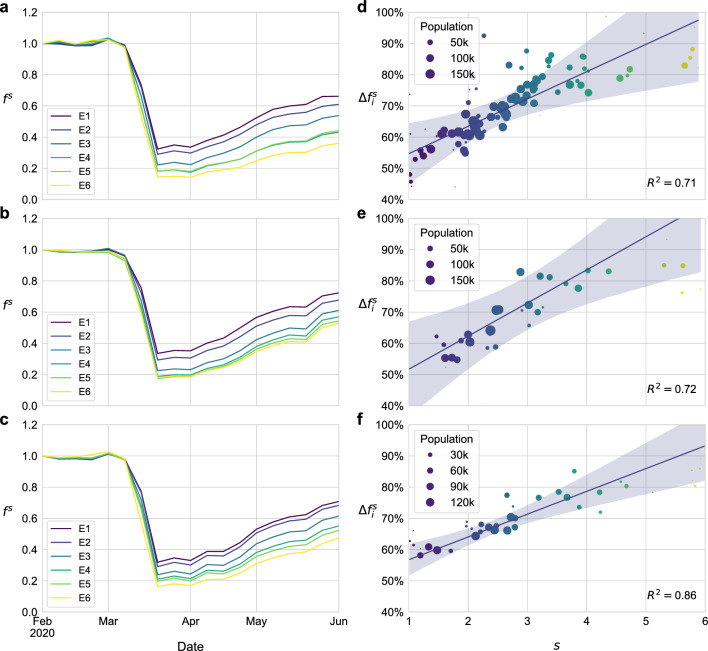
(**a**–**c**) Time evolution of the aggregated number of trips for each stratum (color code), re-scaled to a reference value set on 2020-02-02, corresponding to the pre-pandemic scenario. (**d**–**f**) Reduction in the trips departing from each patch on the week starting from 2020-03-29 compared to a baseline scenario (2020-02-02) according to the average economic stratum of its residents. The size of the dots denotes the number of residents in the corresponding patch whereas the color encodes the stratum information ranging from poorest areas (blue) to the richest ones (yellow). The shaded area is the prediction interval of the linear regression, and it represents the standard error of the predictions from the model, obtained with the member *var_pred_mean* from the Python library *statsmodels*. From top to bottom, the information shown corresponds to Bogotá, Medellín and Santiago de Cali respectively.

### Mobility, lockdown and socioeconomic classes

We first analyze the socioeconomic determinants shaping the relationship between lockdown policies and mobility reduction. To do so, we construct a time-varying mobility network associated with each stratum by merging weekly mobility patterns estimated from mobile phone users with socioeconomic information provided by census surveys as detailed in the “Methods” section. For the sake of illustration, we represent in Fig. 1 the spatial distribution of: the number of residents (a-c), the household stratum (d-f), and the aggregated mobility patterns (g-i) for each of the three cities here analyzed.

**Figure 3:**
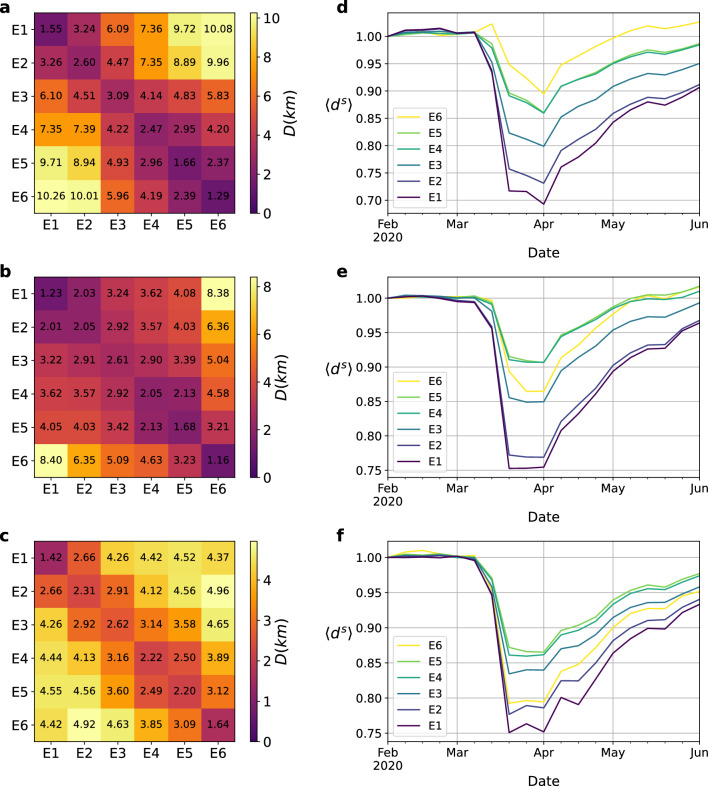
(**a**–**c**) Distance matrices $\textbf{D}$ encoding the average distance of trips departing from areas associated with one stratum (rows) and arriving in going to stratum $s_2$ areas (see “Methods” for an explanation of their computation). These values are computed using mobility patterns from the week starting on 02-02-2020, which corresponds to a pre-pandemic scenario. (**d**–**f**) Time evolution of the average distance of travels made by individuals belonging to each stratum *s*, $\langle {d^s}\rangle$ (color code). Note that the values represented have been re-scaled by those corresponding to the pre-pandemic scenario. From top to bottom, the information shown corresponds to Bogotá, Medellín and Cali respectively.

Once constructed the multiplex mobility network, we can focus on the reduction in the level of mobility of each stratum, which we can calculate by aggregating all the flows in each layer as detailed in “Methods” section. Figure 2a–c showcase the effectiveness of the lockdown measures, as the three cities achieved a significant reduction in the overall mobility of their populations. This, however, was not homogeneous across strata, being much more significant for the wealthiest strata typically gathering socioeconomic classes with highest income. To further quantify this phenomenon, we compute the reduction in the volume of flows by comparing mobility data corresponding to the first week of April and the first of February in each geographical area (patch) as explained in “Methods” section. Figure 2d–f confirm the positive correlation between the average economic stratum in each patch and the reduction in mobility achieved by its residents. This correlation is consistent across the three cities here analyzed, being the Spearman correlation coefficients between both variables $\rho _S=0.832$, $\rho _S=0.878$ and $\rho _S=0.924$ for Bogotá, Medellín and Santiago de Cali respectively.

### Lockdown restructures mobility networks

So far, we have tackled how socioeconomic features shape the impact of lockdown policies on human mobility by studying how the macroscopic volume of movements associated with each stratum varied as these policies were enforced. In this section, we are interested in addressing whether, beyond the overall reduction in the level of mobility across the city, these interventions modified substantially the microscopic structure of the mobility network of each stratum. To do so, we focus on two different indicators: the distance involved in each stratum’s trips and the socioeconomic composition of their destinations.

**Figure 4:**
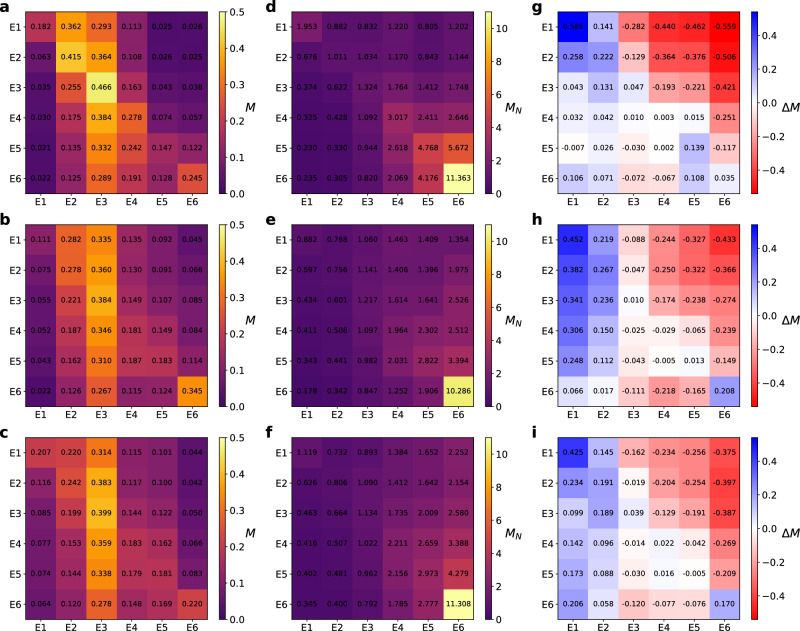
(**a**–**c**) Mixing matrices $\textbf{M}$ encoding the fraction of trips corresponding to a certain stratum (rows) arriving in areas associated with another stratum (columns) on 2020-02-02 (pre-pandemic scenario). The sum of the elements on each row is equal to one. (**d**–**f**) Re-normalized mixing matrices $\mathbf{M_N}$ based on the proportion of the population belonging to each stratum, as detailed in the “Methods” section. In these matrices, entries above (below) one denote a higher (lower) tendency to interact with a given stratum than the one expected in a well-mixed population. (**g**–**i**) Relative change of these matrices $\Delta M$ comparing a lockdown scenario, corresponding to the week starting on 2020-03-29, with a pre-pandemic scenario, corresponding to the week starting on 2020-02-02. From top to bottom, the information shown corresponds to the cities of Bogotá, Medellín and Santiago de Cali respectively.

Before studying the impact of lockdown policies, let us characterize the structure of the mobility networks in the baseline scenario (February 2020) before the arrival of COVID-19 pandemic in the three cities here analyzed. For this purpose, we compute the matrix $\textbf{D}$, where each element $D^{s_1s_2}$ of this matrix is the average distance of the flows from the zones associated with stratum $s_1$ to those associated with stratum $s_2$ (see “Methods” for the computation of the matrix). Figure 3a–c show that the different strata are systematically segregated in the cities studied, since the distance between two strata increases as they become less similar from a socioeconomic point of view. These results, computed from the merged mobility networks, are consistent with the clear segregation observed in Fig. 1d–f showing the spatial distribution of strata across these cities with the finest spatial resolution of census blocks.

To compute the impact of lockdown policies, we represent in Fig. 3d–f the time evolution of the average distance of trips departing from areas associated with each stratum $s_1$, denoted by $\langle {d^{s_1}}\rangle$ (see “Methods” for the derivation of this quantity). In these panels, we observe a robust trend across the three cities consisting in a drastic reduction of trip distances as soon as NPIs were implemented. Note, however, that this effect is not homogeneous across each city, being more pronounced for those trips departing from areas associated with lowest strata.

To shed light into how the heterogeneous trends observed in Fig. 3d–f affect social mixing, we investigate how lockdown policies altered the socioeconomic composition of the destinations visited by each stratum. This information is encoded in the mixing matrix $\textbf{M}$ in which each element $M_{s_1 s_2}$ denotes the average fraction of individuals belonging to stratum $s_2$ in the destinations of the trips departing from stratum $s_1$ areas. The mathematical expression of these elements can be found in the “Methods” sections. As for the distance analysis, let us first analyze the mixing matrix $\textbf{M}$ in the baseline scenario, when no lockdown policies were at play. Figure 4a–c confirm the strong socioeconomic impact on mixing patterns in Colombian cities. Specifically, we observe that trips connecting areas populated by similar strata are overrepresented whereas connections among distant strata are hampered. By removing the bias related to the unequal population size of the different strata and computing the re-normalized mixing matrix $\mathbf{M_N}$ (see “Methods” for details on its calculation), the former result becomes more evident, as illustrated in Fig. 4d–f.

**Figure 5:**
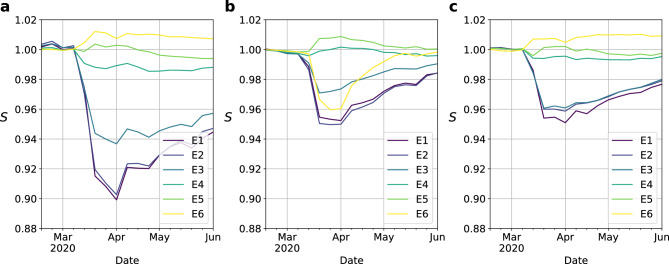
Time evolution of the relative entropy *S* for each row of the mixing matrix, which encodes the socioeconomic structure of the destinations visited by each stratum (color code), in Bogotá (**a**), Medellín (**b**) and Cali (**c**) respectively. Note that the values represented have been re-scaled to those corresponding to the week starting on 02-02-2020, corresponding to a pre-pandemic scenario. Let us note that lower entropy values reflect more concentrated flows towards a small number of economic strata.

In Fig. 4g–i we show how lockdown policies altered the elements of the mixing matrices. There, by comparing the structure of the matrices corresponding to the baseline pre-pandemic scenario and the week starting from 29th March (during lockdown), we observe that the impact of the implemented policies on the structure of mobility patterns is quite heterogeneous across strata. Once again, these heterogeneities across strata are consistent in the three cities here studied. In the first place, confinement policies provoked important modifications in the mobility patterns of lowest strata, exacerbating their endogenous nature while making it difficult for them to mix with those belonging to higher strata. Taking this result together with the average trip distance connecting different strata shown in Fig. 3, we realize that the pronounced drop in the trip distance for the lowest strata is explained by the huge reduction in long-distance trips connecting them to areas populated by the higher strata. In contrast, lockdown policies seemed to have a less relevant impact on the structure of contacts of the wealthiest strata in Colombia. However, note that, despite this overall small variation, lockdown policies appeared to increase the diversification of their mobility patterns, as they moved more evenly to places belonging to socially distant individuals.

To round off our study, we compute the time evolution of the Shannon entropy of the entries of the mixing matrices corresponding to each stratum and represent them in Fig. 5. The entropy values shown in these figures are normalized by their values as of 02-02-2020 (before the NPIs came into force). The three panels confirm the previous results: lockdown policies reduced considerably the social variability of the destinations visited by lowest strata whereas it left almost unaltered those corresponding to highest ones. However we find an exception in Medellín, where population from stratum 6 tend to isolate more as also shown by Fig. 4h.

## Discussion

The extremely rapid unfolding of COVID-19 pandemic at the beginning of 2020 arose from the subsequent combination of the international exportation of infectious cases^35,36^ through the airport mobility network and the local exponential growth in the number of cases driven by community transmission of the virus. Since vaccines were not available at the time, many countries deployed NPIs to reduce the impact of the pandemic. These containment measures included mobility restrictions to curb the importation of cases from high-incidence areas and local control policies that reshaped the contact patterns of the population responsible for community transmission of the virus.

In this work, we have studied how NPIs altered the mobility patterns of different socioeconomic classes in the three main cities of Colombia. Our analysis reveals a strongly unequal impact of control policies on the different strata into which Colombian society is divided. First, we have reported a consistent positive correlation between the reduction in the number of trips registered for a given area and the average stratum of its residents, implying that those individuals with more economic resources, typically belonging to the highest strata, were more able to reduce their mobility and, therefore, their social activity. Secondly, we have revealed that lockdown policies, in addition to affecting the volume of movements recorded in each city, modified their architecture, reducing the distance involved in the movements and increasing the social similarity in the composition of the origin and destination of the flows recorded. However, this phenomenon was not homogeneous in all strata. The blocking policies hindered long-distance urban trips connecting lowest socioeconomic classes with wealthier areas, leading to a significant increase in the endogenous interactions with geographically proximate individuals and a reduction in the distance of their trips. In contrast, our analysis reveals that the implemented policies hardly affected the mobility patterns of the highest strata.

Our results suggest the need for accounting for socioeconomic variables when assessing the feasibility or the expected impact of community NPIs to mitigate an epidemic outbreak. In this sense, despite the clear improvement of the health situation while gaining time for vaccine development, the socioeconomic dimension of NPIs entailed undesired collateral effects such as economic crisis^37,38^, mental health issues^39–45^, or the increase of social inequalities in different countries as a result of lockdown policies^46–48^. Beyond social implications, the exacerbation of social inequalities might also have important implications for the evolution of epidemic outbreaks, as proven by the influence of income gradients on COVID-19 associated mortality^49–51^. Apart from NPIs, socioeconomic determinants are also crucial to understand other important factors during COVID-19 pandemic such as the access to health services^52–54^, the unequal vaccination willingness observed in the population^55–57^ or the heterogeneous occurrence of mental health issues across social and age groups^58^. We hope that our study will shed light into the interplay between socioeconomic information and epidemic spreading and will pave the way to the design of control policies optimizing the trade-off between their health outcome and the damage of the socioeconomic fabric derived from their implementation.

### Limitations

Our results should be interpreted in light of several limitations. First, the Google mobility data is limited to smartphone users who have opted in to Google’s Location History feature, which is off by default. Therefore, we assume the mobility patterns of this set of individuals to be representative of the movements of the entire population. Importantly, these limited data are only viewed through the lens of differential privacy algorithms, specifically designed to protect user anonymity and obscure fine detail. Furthermore, comparisons across rather than within locations are only descriptive since these regions can differ in substantial ways.

Another limitation of this data set is that the origin of one movement recorded for one individual does not necessarily coincide with its residence, which might introduce some noise when computing the reduction of the mobility associated to each stratum. Nonetheless, previous studies leveraging this kind of data sets have found comparable results to those obtained when analyzing mobility patterns coming from census surveys^18,33^.

Moreover, to construct the multiplex mobility networks, we have assumed that all socioeconomic strata are proportionally represented in the data set, thus neglecting socioeconomic biases in the use of mobile phone devices, which may vary by location. While this assumption could remain controversial, our results are consistent with those obtained by analyzing variations in public transport usage as a result of lockdown policies^32^.

Finally, we have restricted our analysis to cities in Colombia, where socioeconomic classes are highly segregated. This segregation is partly explained by the spatial distribution of these populations across cities, where poor populations tend to be located on informal settlements along the cities’ boundaries. Frequently, these outskirts tend to be more disconnected from city centers and economical hubs due to scarce access to transport services. The generality of our findings when extending our analysis to other societies deserves further investigation and remains as future work.

## Data Availability

Mobility data are extracted from the Google SARS-CoV-2 Aggregated Mobility Research Dataset and are available with permission from Google LLC. Data regarding the demographic and socioeconomic information are publicly available and provided by the national statistics department in Colombia (accessed 2023-07-21, https://www.dane.gov.co/index.php/estadisticas-por-tema/demografia-y-poblacion/censo-nacional-de-poblacion-y-vivenda-2018). Data about the time evolution of COVID-19 cases are extracted from official data reported by the authorities in Colombia (accessed 2023-02-14, https://www.datos.gov.co/Salud-y-Protecci-n-Social/Casos-positivos-de-COVID-19-en-Colombia/gt2j-8ykr/data). The datasets used and/or analysed during the current study are available from the corresponding author on reasonable request.

## Code availability

Codes used to generate the figures of the manuscript are available from the corresponding author on reasonable request.
